# Exploring the Influence of Inter-Trial Interval on the Assessment of Short-Interval Intracortical Inhibition

**DOI:** 10.3390/bioengineering11070645

**Published:** 2024-06-25

**Authors:** Lidio Lima de Albuquerque, Milan Pantovic, Erik W. Wilkins, Desiree Morris, Mitchell Clingo, Sage Boss, Zachary A. Riley, Brach Poston

**Affiliations:** 1 School of Health and Applied Human Sciences, University of North Carolina Wilmington, Wilmington, NC 28403, USA; limadeal@uncw.edu; 2 Health and Human Performance Department, Utah Tech University, St. George, UT 84770, USA; milan.pantovic@utahtech.edu; 3 Department of Kinesiology and Nutrition Sciences, University of Nevada Las Vegas, Las Vegas, NV 89154, USA; wilkie1@unlv.nevada.edu; 4 School of Medicine, University of Nevada-Las Vegas, Las Vegas, NV 89154, USA; morrid3@unlv.nevada.edu (D.M.); clingom@unlv.nevada.edu (M.C.); 5 School of Life Sciences, University of Nevada-Las Vegas, Las Vegas, NV 89154, USA; bosss1@unlv.nevada.edu; 6 Department of Kinesiology, Indiana University Purdue University Indianapolis, Indianapolis, IN 46202, USA; zariley@iupui.edu

**Keywords:** short-interval intracortical inhibition, transcranial magnetic stimulation, motor evoked potential, intracortical facilitation, short-interval intracortical facilitation, electromyography

## Abstract

Short-interval intracortical inhibition (SICI) is a common paired-pulse transcranial magnetic stimulation (TMS) measure used to assess primary motor cortex (M1) interneuron activity in healthy populations and in neurological disorders. Many of the parameters of TMS stimulation to most accurately measure SICI have been determined. However, one TMS parameter that has not been investigated is the time between SICI trials (termed inter-trial interval; ITI). This is despite a series of single-pulse TMS studies which have reported that motor evoked potential (MEP) amplitude were suppressed for short, but not long ITIs in approximately the initial ten trials of a TMS block of 20–30 trials. The primary purpose was to examine the effects of ITI on the quantification of SICI at rest. A total of 23 healthy adults completed an experimental session that included four SICI trial blocks. Each block utilized a different ITI (4, 6, 8, and 10 s) and was comprised of a total of 26 SICI trials divided into three epochs. ANOVA revealed that the main effects for ITI and epoch as well as their interaction were all non-statistically significant for SICI. We conclude that the shorter (4–6 s) ITIs used in studies investigating SICI should not alter the interpretation of M1 activity, while having the advantages of being more comfortable to participants and reducing the experimental time needed to evaluate perform single and paired-pulse TMS experiments.

## 1. Introduction

Transcranial magnetic stimulation (TMS) is an established noninvasive brain stimulation technique that is frequently used to evaluate net corticospinal excitability in neurophysiology and motor control studies [1,2,3,4,5]. This is accomplished through the measurement of the motor evoked potential, which is the brief response generated in the electromyography (EMG) recording about 21–23 ms after a single TMS pulse above motor threshold is delivered to the primary motor cortex (M1) [3,6]. Moreover, two separate MEPs can be combined in short succession in paired-pulse TMS protocols. This involves the application of a below-threshold conditioning TMS pulse that is followed by an above-threshold test TMS pulse using various timespans (inter-stimulus intervals; ISIs) between the conditioning and test pulses. In addition, the exact stimulation intensities of the pulses can also be modulated. Accordingly, a number of condition–test protocols have been developed to quantify different intracortical interneuronal inhibitory and excitatory pathways in M1 [7,8,9]. These primarily include short-interval intracortical inhibition (SICI), intracortical facilitation (ICF), long-interval intracortical inhibition, and short-interval intracortical facilitation (SICF). Importantly, extensive prior combined physiological and pharmacological studies have determined that the function of these pathways are mediated by different neurotransmitter and receptor systems. However, SICI is the most extensively investigated of these intracortical neuron systems [7,10,11,12], and seems to have the most direct relevance to certain aspects of motor behavior, such as movement initiation [13], force relaxation [14], and motor learning [15,16,17].

The phenomenon of SICI was first identified in a classic study [10] by Kujirai and colleagues (1993). The research methodology was based on older animal studies that utilized cortical electrical stimulation and involved interhemispheric inhibition [18]. The main findings were that short ISIs of 1–6 ms resulted in pronounced inhibition of the condition–test MEP compared to the test MEP alone. In contrast, longer ISIs of 10 and 15 ms resulted in pronounced facilitation of the condition–test MEP compared to the test MEP alone. These measurements later became to be known as SICI and ICF, respectively. Subsequently, research focused on the best stimulation intensities and ISIs to employ to optimize the measurement of SICI with a study by Ortu and colleagues probably representing the most comprehensive SICI parameter assessment [12]. Extensive work has also been performed to uncover the physiological mechanisms underlying SICI and the roles that it may play in movement. SICI results from the activation of an inhibitory population of small inhibitory neurons that modulate the activity of pyramidal cells. Specifically, accumulated evidence from concurrent cortical physiology and spinal reflex measurements as well as studies involving descending spinal cord volleys determined that SICI originates at the cortical and at the levels of the brainstem or spinal cord [7,11,13,19]. In addition, SICI has been determined to be primarily mediated via chandelier cell activity through inputs onto M1 pyramid cells at their axonal hillock. Furthermore, pharmacological studies have pointed to the involvement of the alpha-2-subunit-bearing subtype and not the alpha-1-subunit-bearing subtype of the GABA_A_ receptor in these connections. The interneuronal populations responsible for SICI also receive inputs from other intercortical and intracortical pathways (for visual depictions and review, see Reis et al., 2008 [13]). The functional significance of these pathways to and from SICI has been demonstrated by a observations that SICI is not only involved in the initiation of movements, force modulation, and motor skill acquisition in healthy adults, but is also impaired in a number of movement disorders [7,11,19] that exhibit deficits in these and other facets of movement control.

Based on the widespread application and critical role TMS plays in many neurophysiology and motor control studies, considerable efforts have been dedicated to establishing optimal methodological frameworks [20] and formulating guidelines published in review articles for conducting TMS research utilizing these approaches [11]. An example of such an effort was the convening of a large international expert panel where the relative importance of over 20 critical methodological aspects were surveyed [21]. The time between MEP trials, henceforth referred to as the inter-trial interval (ITI), and not to be confused with ISI, was included as one of these critical TMS parameters. The results revealed that over 80% of the panelists voted that ITI was important or very important to experimentally control and that it’s chosen value should be explicitly stated always or most of the time. This is notable, as this relatively early paper is perhaps the only review that has dedicated any attention to ITI. In spite of this consensus viewpoint and other strong evidence from other types of stimulation such as the H-reflex [22,23] and auditory evoked potentials [24], there seem to be no specific standards concerning ITI to implement between single-pulse and especially paired-pulse MEPs. This is in stark contrast to numerous studies and reviews concerning almost all of the other 20 aforementioned methodological items involved in TMS studies. It is therefore not surprising that a large and varied assortment of ITI approaches are present in the literature. For example, some studies have used exceedingly long ITIs ranging from 15 to 30 s [25,26], whereas others have used exceptionally short ones of between 1.5 and 5 s [12,27]. Another practice is to vary the ITI between trials randomly within range around a set average ITI [28,29]. Additionally, it appears that the most frequently employed ITIs span between 4 or 6 s, though a significant portion of studies either use ambiguous wording or neglect to report the ITI altogether [30].

The limited focus on ITI in comparison to other TMS stimulation parameters is not easily explained, but likely stems from the prevailing assumption that only repetitive TMS techniques involving high-frequency stimulation over extended periods result in lasting post-stimulation effects [31,32,33]. Accordingly, ITIs of one second or more would be expected to not induce time-dependent changes in MEP amplitudes across successive trials. This view is supported by findings suggesting that single TMS pulses to M1 transiently enhance cortico-muscular coherence for only a brief period of less than a second before returning to pre-stimulation levels [34]. However, certain studies contradict this notion, showing variations in MEP amplitudes within a series of trials, indicating potential influences of ITI on single-pulse TMS MEP measurement [35]. Another more comprehensive study demonstrated statistically significant differences in MEPs evoked at short ITIs (5 s and below) versus a long (10 s) ITI using a set TMS stimulation intensity. Most importantly, this phenomenon mainly occurred in the first 10 of the 30 MEPs constituting a block of trials. These studies and others [36,37] suggest that ITI may indeed play a non-trivial role in the variability of MEP measurements, which would warrant further investigation and potential reevaluation of ITI practices in TMS research to ensure valid and reliable findings.

A recent study performed in our laboratory was the first to determine the influence of ITI on any measure of paired-pulse TMS [38] and found no effect of ITI on ICF. However, no studies have explored how different ITIs might affect quantification of SICI. This is despite the prevalence of SICI research, the importance of SICI in movement control, and that SICI is mediated by different neurotransmitter and receptor systems than other paired-pulse TMS measures such as ICF. Furthermore, single-pulse TMS seems to be subject to the influence of ITI [35,36,37,39,40,41] and SICI quantification involves the pseudorandom interleaving of condition–test MEP trials and single-pulse test MEP trials within a TMS block. In addition, a test MEP is also obviously a constituent of the condition–test MEP. These lines of rationale form the basis for the possibility that short ITIs of approximately 6 s and below that have been used in the majority of SICI studies may have negatively affected the SICI values obtained in studies involving healthy adults and in patients with movement disorders. This possibility would be more likely the many studies that have involved small sample sizes or a relatively small number of MEP trials [42], which have also been the case in many SICI studies. Thus, the primary purpose was to examine the effects of ITI on the quantification of SICI at rest. This was accomplished by quantifying SICI in four separate trial blocks that utilized ITIs of 4, 6, 8, and 10 s. Based on a number of previous single-pulse TMS studies [35,36], it was predicted that the magnitude of SICI in the short (4 s ITI) SICI block would be increased (greater inhibition) compared with the 6, 8, and 10 s ITI SICI blocks. In addition, it was hypothesized that these differences would be manifested through an initial suppression (greater inhibition) over approximately the first 8–10 MEP trials and would not be due to a serial increase (more inhibition) in SICI over the entire trial block. The secondary purpose was to determine the effects of ITI on the quantification of single-pulse MEP amplitudes at rest. This was achieved by measuring single-pulse MEP amplitudes in two control blocks using ITIs of 4 and 10 s, respectively. It was predicted based on prior single-pulse TMS studies [35,36,37] that the 4 s ITI control block would exhibit a lower average MEP amplitude compared with the 10 s ITI control block. Finally, it was expected that this would be due to significantly lower MEP amplitudes over approximately the first 8–10 MEP trials and not a gradual decrease in MEP amplitude over the entire duration of the 4 s ITI control block.

## 2. Materials and Methods

### 2.1. Participants

The experiments were performed on the right hand of a total of 23 (12 males and 11 females; average age: 26.7 ± 6.0 years) healthy young participants. Participants were recruited using flyers posted in several buildings throughout the university. Inclusion criteria included (1) ability to provide informed consent; (2) being free from any known neurological or psychiatric condition; (3) age between 18 and 45 years old; and (4) being right-handed. Accordingly, all participants were right-handed as evidenced by the Edinburgh Handedness Inventory [43]. Exclusion criteria included (1) an uncontrolled medical condition; (2) metal in the skull or eye such as a cardiac pacemaker, brain stimulator, shrapnel, surgical metal, clips in the brain, cochlear implants, and metal fragments in the eye; (3) diagnosed hearing loss; (4) having had a brain tumor, a stroke, head trauma, epilepsy, or a history of seizures, having a neurological disorder or a movement disorder, or having a head injury that involved being passed out for more than a few seconds; and (5) being pregnant or thought to be pregnant. Finally, participants were screened to confirm that they did not meet the exclusion criteria for noninvasive brain stimulation [20]. The study was approved by the Institutional Review Board at the University of Nevada, Las Vegas and performed in accordance with the Declaration of Helsinki.

### 2.2. Experimental Design

**Figure 1:**
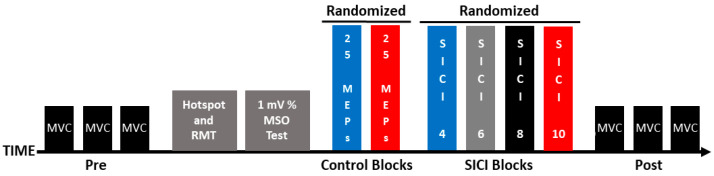
Experimental protocol. Each experiment involved the following set of procedures: pre-MVCs, identification of the motor hotspot location, RMT determination, 1 mV stimulation intensity quantification, control blocks (1 mV_4 and 1 mV_10), SICI blocks (SICI_4, SICI_6, SICI_8, and SICI_10), and post-MVCs.

Participants completed a single experimental session (~2 h), and the experimental steps were completed in the following order: (1) maximum voluntary contractions (MVCs); (2) identification of the motor hotspot location; (3) resting motor threshold (RMT) determination; (4) 1 mV stimulation intensity quantification; (5); two control blocks of single-pulse MEPs that were evoked with ITIs of 4 and 10 s (hereafter referred to as the 1 mV_4 and 1 mV_10 conditions); (6) four SICI trial blocks that involved ITIs of 4, 6, 8, and 10 s (hereafter referred to as the SICI_4, SICI_6, SICI_8, and SICI_10 conditions), and (7) MVCs. These experimental steps are depicted in Figure 1 and the methodology of each of the steps is described in the sections below.

### 2.3. Experimental Arrangement

The first dorsal interosseus (FDI) muscle of the right hand was the target muscle for all experimental testing. Participants sat in a chair beside a table with the forearm resting on the table surface. The upper limb posture was set so that the shoulder was abducted (~45 degrees), the elbow was flexed (~90 degrees), the wrist was in neutral, and the right hand was prone [44]. This posture was strictly maintained during all TMS procedures as it has been clearly shown that MEPs evoked in hand muscles can be significantly altered when the configuration of the upper limb is changed [45,46]. The right FDI muscle EMG activity was provided as feedback on a computer monitor that was situated in front of the participants. The participants were given stringent and detailed directions before the TMS testing blocks began on how to utilize the visual EMG feedback to make sure that the FDI was deactivated and at rest for all of the TMS testing procedures. In all TMS testing, the participants could see the baseline EMG noise level on the screen at a high gain [47]. A black horizontal cursor line was placed at a level of 25 microvolts above the top of the baseline noise. Participants were told not to let the EMG level go above this line for a sustained period of time as it would represent a light muscle contraction. To further ensure that the FDI was relaxed, the same computer display was continually monitored by one of the three to four investigators present in the data collection of each experiment. This investigator had the sole responsibility of incessantly scrutinizing the EMG level and the participant’s body and hand posture. Accordingly, this investigator provided verbal feedback to participants as needed if the EMG signal indicated that the FDI was contracting at any time point during the TMS testing blocks. Finally, the data analysis programs checked for any MEP trials that had an FDI EMG level greater than an average of 25 microvolts in the 50 ms before the MEP was evoked in each trial. This was carried out to identify trials for further inspection and rejection if this criterion were met, which is a common process in TMS experiments [48]. However, no trials had to be rejected in the study.

TMS was applied via two Magstim 200^2^ stimulators linked by a Bistim module and through a double 70 mm remote control figure-of-eight coil. The TMS unit was put in the Bistim Mode [49] configuration for all of the single and paired-pulse TMS measurements. The coil was positioned tangential to the scalp and the coil handle was positioned laterally and backwards at an angle of 45 degrees with respect to the midline. The TMS coil was placed over the scalp site corresponding to the FDI muscle’s “motor hot spot” of the left M1 to elicit MEPs in the FDI of the right hand [50]. The right FDI EMG activity was recorded with surface electrodes placed in a belly tendon montage. The EMG signals were collected utilizing Cambridge Electronic Design (CED); Cambridge, UK software (Signal 5.04) and hardware (micro 1401 data acquisition interface and 1902 amplifiers).

### 2.4. Experimental Procedures

#### 2.4.1. MVCs

The MVCs were performed using index finger abduction as the FDI was the target muscle for the TMS procedures and almost all index finger abduction force is generated by the FDI muscle [51]. A custom manipulandum mounting a force transducer was located on the table close to the block where the hand was placed. This allowed participants to produce force on that transducer at the proximal interphalangeal joint of the right index finger. The MVCs were collected using methodology similar to prior studies [52,53,54]. Participants were required to exert their maximum force in the shortest possible time and to maintain the maximum for approximately 5 s [53]. The FDI force was provided to participants by a red force trace on a computer monitor. A total of three MVC trials were performed at both the start (pre-MVCs) and end of the experimental session (post-MVCs) with a minute of rest between all trials.

These pre- and post-MVCs were completed to give some assurance that the voluntary activation capacity of the right FDI muscle had not considerably decreased during the experimental session due to some manifestation of central or mental fatigue. For example, MEP amplitude can be affected by changes in attention, arousal, and alertness [6]. Since the experiments in this study lasted approximately two hours, the levels of concentration needed during the experiment could potentially have led to mental fatigue and influenced MEP measurements. Accordingly, it has been established since the beginning of fatigue research that voluntary muscle activation can be reduced following mental fatigue alone [55]. While the probability of meaningful levels of mental and central fatigue were likely very low due to all of the experimental procedures being completed with the FDI muscle at rest, the MVCs nonetheless served as a relevant, simple, and time-efficient experimental control.

#### 2.4.2. Motor Hotspot Identification

The TMS coil was moved over the scalp while suprathreshold TMS pulses were applied until the point where the highest MEPs in the right FDI was identified. This site was designated as the FDI motor hot spot, and all MEPs were evoked from this location. Finally, the associated TMS coil position was outlined on a scalp cap to ensure a constant coil position throughout the experiment and the position of the scalp cap on the forehead was marked using an erasable marker [50].

#### 2.4.3. RMT

RMT was quantified according to standard practice and was defined as the lowest TMS stimulation intensity needed to elicit a MEP with a 50-microvolt peak-to-peak amplitude in a minimum of 5 out of 10 consecutive TMS trials [56]. The RMT obtained from each participant was taken to calculate the individual TMS conditioning pulse stimulation intensity needed for the SICI measurements.

#### 2.4.4. The 1 mV Stimulation Intensity Quantification

The 1 mV stimulation intensity as a percentage of maximum stimulator output (% MSO) was determined according to the procedures of previous studies [57,58]. Briefly, the stimulation intensity started at 55% of MSO and adjusted while MEPs were monitored and quantified online until the average MEP amplitudes were as close as possible to 1 mV. Subsequently, this stimulation intensity was utilized for all of the single-pulse TMS MEPs obtained in the control blocks and for the test MEPs in the SICI blocks. Importantly, the 1 mV stimulation intensity quantification was completed using an ITI of 10 s as this corresponded to the longest ITI in the present study. In addition, this choice was based on prior single-pulse TMS ITI studies [35,36,37] whose results had collectively indicated that ITIs of 10 s and above should definitely be sufficient to reflect the best estimate for 1 mV stimulation intensity value and should not be subject to time-dependent effects.

#### 2.4.5. Control Blocks

Two separate control blocks involving single-pulse TMS trials were completed in randomized order. These blocks were included in the study so that the findings could be compared to a series of prior ITI studies [35,36,37] that only involved single-pulse TMS. In addition, these blocks also provided a control comparison to the single-pulse test MEPs evoked in the SICI blocks. One control block utilized an ITI of 4 s, whereas the other control block utilized an ITI of 10 s (herein referred to as the 1 mV_4 and 1 mV_10 control blocks, respectively). Therefore, the control blocks comprised the shortest (4 s) and longest (10 s) ITIs that were later employed in the SICI blocks. Since differences across ITIs were most likely to be seen between the 4 and 10 s ITIs, the 6 and 8 s ITIs used in the SICI blocks were not included in the control blocks to keep the total experiment time from exceeding two hours.

Both control blocks consisted of 25 MEPs evoked utilizing the previously determined 1 mV stimulation intensity for each individual participant. The 1 mV stimulation intensity is the most common value used to quantify changes in MEP amplitude before and after various experimental interventions in TMS studies. Furthermore, the stimulation intensity to evoke a 1 mV MEP also serves as the test MEP stimulation intensity in virtually all paired pulse TMS studies. The total of 25 MEP trials per block was chosen based on the following rationale. First, a detailed quantitative study [42] reported that a total of 20–30 MEPs per trial block strikes the best balance between the requisite number of trials to minimize estimation error for average MEP. Second, this was also the range of MEPs that the same authors determined is most appropriate and realistic to accomplish due to time and other constraints inherent in most TMS studies; and third, the most comparable previous ITI studies involving single-pulse TMS utilized 25 and 30 MEP trials per block [35,37].

#### 2.4.6. SICI Blocks

SICI was measured at ITIs of 4, 6, 8, and 10 s (herein referred to as SICI_4, SICI_6, SICI_8, and SICI_10) in four separate blocks of trials that were completed in randomized order. Thus, the only difference between the SICI blocks was the ITI that was utilized. SICI was evoked using constant parameters that were selected because they have been the most common in the literature and determined to be the optimal to detect SICI at rest [12]. Specifically, the conditioning pulse stimulation intensity was 90% of RMT, the test pulse stimulation was the 1 mV stimulation intensity as a % MSO, and the ISI was 3 ms.

Each of the four SICI blocks consisted of 52 total trials that included 26 test MEP trials evoked with single-pulse TMS and 26 condition-test MEP trials evoked with paired-pulse TMS. These two types of TMS trials were delivered in a semi-randomized order, which involved each consecutive set of two TMS trials being randomized between the two types of trials. Therefore, a total of 26 SICI measurements were taken in each block. SICI was calculated by dividing the condition–test MEP amplitude by the test MEP amplitude and expressed in percentage terms, which corresponds to the percent inhibition. Twenty-six trials per block were chosen for reasons analogous to those stated above for the number of 25 total MEPs in each of the control blocks. Furthermore, this would have permitted a minimum of 25 SICI measurements in case the first test MEP and condition–test MEP would have had to be deleted from the analyzes. However, this deletion ultimately was not necessary as the first 1–2 MEP trials were similar in amplitude to the average of all the other trials within each trial block (see Section 4.3 of the Discussion).

The specific ITIs of 4, 6, 8, and 10 s utilized for the SICI blocks were selected according to the following rationale: (1) While a few of the prior studies that investigated the influence of ITI on single-pulse MEP amplitude used ITIs of less than four seconds [35,40], extensive pilot testing revealed that ITIs of less than four seconds was not always feasible for SICI testing. In these conditions, the TMS unit would sometimes skip trials as a result of the capacitors not recharging fast enough if a given participant had a high RMT and 1 mV MEP, which would correspond to the need for relatively high stimulation intensities for the test MEPs and condition–test MEPs. Similarly, evoking SICI with ITIs of less than four seconds also resulted in coil overheating for a nontrivial number of participants. In the current study, these issues would have rendered the results meaningless since the change SICI on a trial-by-trial basis as a function of time was a primary interest. (2) Based on prior single-pulse TMS studies involving ITI, the shortest 4 s ITI should be brief enough to be able to identify any time-dependent modulations in MEP amplitudes due to ITI, if they were to exist [35,36,37]. Specifically, these studies demonstrated that ITIs of 5 s and below influenced MEP amplitudes. Relatedly, these studies clearly indicated that a 10 s ITI would be sufficient to provide valid and reliable MEP amplitude measurements given that ITIs as low as approximately 6–8 s and especially 10–20 s were not subject to time-dependent effects; (3) it quickly became evident that ITIs of greater than 10 s would not be practical for almost all TMS experiments. The total time required to conduct TMS experiments would be far too great, the experiments would be too unpleasant for the participants and investigators, and the number of blocks, conditions, and total trials would have to be reduced compared to what is usually desired in most TMS experiments. In summary, both prior research and extensive piloting deemed that the shortest ITI of 4 s should be adequate to find time-dependent effects and the longest ITI of 10 s should be more than long enough to provide valid and reliable measures of MEP amplitudes to compare to the shorter ITIs.

### 2.5. Data Analysis

The MVC and MEP data were reduced and analyzed using custom Signal software scripts by members of the research team who were not present during data collection. Accordingly, the investigators who collected the data during the experimental sessions did not perform the data analysis [59].

#### 2.5.1. MVC Force, MVC EMG, RMT, and 1 mV Stimulation Intensity Analyses

The MVC force was quantified as the average force generated over the plateau portion (usually about three to five seconds) of the MVC trials. The MVC trial with the greatest force for each group of three pre- and post-MVC trials was denoted as the MVC force and used for analysis [44,60]. Similarly, the average FDI EMG was quantified over the same plateau period and the greatest FDI EMG for group of three pre- and post-MVC trials was denoted as the maximum FDI EMG. For all MEP analyses, the MEP size was quantified as the peak-to-peak amplitude value for each MEP. The RMT and 1 mV stimulation intensity (% MSO) are expressed as the averages of the entire sample of participants.

#### 2.5.2. Control Block Analyses

MEP amplitudes were evaluated in three different ways in the control blocks: (1) to assess potential variations in MEP amplitude throughout the control blocks, the 25 MEP trials within each block were divided into three distinct time periods consisting of consecutive MEP trials (epoch 1: trials 1–8; epoch 2: trials 9–16; epoch 3: trials 17–25). As a result, epochs 1–2 included 8 trials each, whereas epoch 3 consisted of 9 trials. This mirrors the approach in our previous investigation on the influence of ITI on ICF [38]. In addition, a similar strategy was used in a single-pulse TMS study, which segmented 30 MEP trials into three equal sub-blocks of 10 MEPs [35]. The decision to allocate 8 trials to epochs 1 and 2 with 9 in epoch 3, as opposed to distributing an equal number of trials across all epochs, accounted for the possibility of excluding the initial trial in each block, a contingency discussed further in Section 4.3 of the Discussion. Nevertheless, this deletion ultimately was not necessary as the first 1–2 MEP trials were similar in amplitude to the average of all the other trials within each trial block. (2) MEP amplitude was also calculated as the average of all 25 MEP trials in each control block to provide a comprehensive view of the average MEP amplitude during control blocks. (3) To examine changes in MEP amplitude across the control blocks in more detail, the average MEP amplitudes for all participants were determined for each of the 25 trials, serving primarily to graphically depict the time series of MEP trials within each control block.

#### 2.5.3. SICI Block Analyses

MEP amplitudes in the SICI blocks were also analyzed in three ways: (1) MEP trials were segmented into epochs, with epochs 1, 2, and 3 comprising 16, 16, and 20 trials, respectively. Beyond the evident rational that these blocks having more total trials due to them involving paired-pulse TMS, the primary justification for the variation in the number of trials across epochs aligns with the previously mentioned rationale concerning the potential exclusion of the initial two trials, which ultimately was not implemented (refer to Section 4.3 of the Discussion). Consequently, the average MEP amplitudes for test MEP trials, condition-test MEP trials, and thus SICI quantification were determined based on the average of 8, 8, and 10 trials for each metric (test MEPs, condition–test MEPs) within the SICI segments and utilized for subsequent analysis. Hence, SICI was determined by division of the condition–test MEP amplitude block average by the test MEP amplitude block average and reporting the as a percentage [7,10,11,12]. (2) The average MEP amplitudes across all 26 test MEP trials, 26 condition–test MEP trials, and thus 26 SICI assessments were compiled for analysis. This was carried out to provide a comprehensive view of the average MEP amplitude during the SICI blocks; (3) to more effectively depict variations in test and condition–test MEP trials across the duration of the SICI blocks, the average test MEP and average condition–test MEP amplitudes for each of the respective trials of all participants was quantified and depicted. This approach was employed for the purposes of illustration and depict the time series of MEP trials within each SICI block.

### 2.6. Statistical Analysis

#### 2.6.1. MVCs

A paired *t*-test was used to compare the pre-MVC and post-MVC force values. Similarly, the pre-MVC EMG and post-MVC EMG values were also compared with a paired *t*-test.

#### 2.6.2. Control Blocks

A 2 *ITI* (1 mV_4, 1 mV_10) × 3 *Epoch* (1, 2, 3) within-subjects ANOVA was used to analyze differences in MEP amplitudes over the time course of the control blocks. To locate where significant differences occurred between pairs of means, post hoc analyses using Bonferroni adjustment for multiple comparisons were performed if appropriate.

#### 2.6.3. SICI Blocks

Three separate 4 *ITI* (SICI_4, SICI_6, SICI_8, SICI_10) × 3 *Epoch* (1, 2, 3) within-subjects ANOVAs were used to analyze the dependent variables of test MEP amplitude, condition-test MEP amplitude, and SICI.

Significance level for all statistical tests was *p* < 0.05, unless modified by Bonferroni corrections. Data are expressed as mean ± standard deviation within the text and mean ± standard error in the figures.

## 3. Results

The mean RMT for the participants was 46.0 ± 9.0 (% MSO), whereas the mean 1 mV stimulation intensity was 59.5 ± 14.1 (% MSO).

### 3.1. MVCs

The paired *t*-test revealed that the difference between the pre-MVC (44.2 ± 10.0 N) and post-MVC force (46.8 ± 12.8 N) values was non-statistically significant (*p* = 0.107, *d* = 0.351). In addition, another paired *t*-test revealed that the difference between the pre-MVC EMG (0.88 ± 0.3 mV) and post-MVC EMG (0.89 ± 0.4 mV) values was non-statistically significant (*p* = 0.753, *d* = 0.066).

### 3.2. Control Blocks

**Figure 2:**
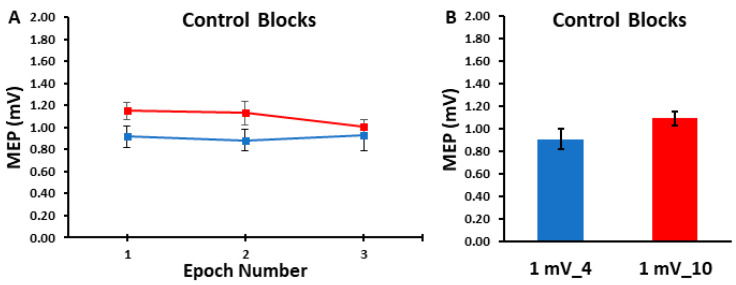
The MEP amplitudes obtained in the control blocks are shown across epoch number (**A**) and as the overall control block averages (**B**). The 1 mV_4 control block (blue) and the 1 mV_10 (red) control blocks displayed statistically similar MEP amplitudes across the three epochs and therefore over each entire trial block.

**Figure 3:**
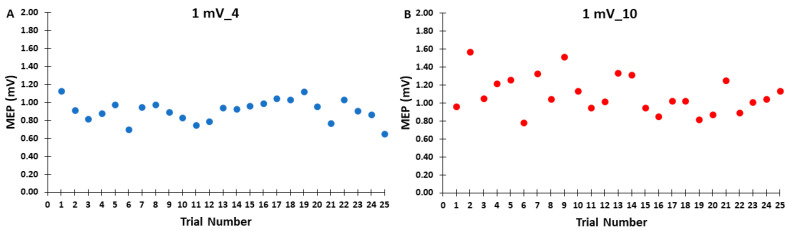
The MEP amplitudes across trials for the 1 mV_4 (**A**) and 1 mV_10 (**B**) control blocks are shown for illustrative purposes, with each data point corresponding to the average MEP amplitude of all participants for each trial in a given control block.

The differences in MEP amplitudes were compared across ITIs and epochs in the control blocks with a 2 *ITI* (1 mV_4, 1 mV_10) × 3 *Epoch* (1, 2, 3) within-subjects ANOVA. The main effect for *ITI* (*p* = 0.103, η_p_^2^ = 0.116), the main effect for *Epoch* (*p* = 0.727, η_p_^2^ = 0.014), and the *ITI* × *Epoch* interaction (*p* = 0.444, η_p_^2^ = 0.036) were all non-statistically significant (Figure 2A,B). Accordingly, Figure 3A,B illustrate that the MEP amplitudes did not show a trend for an increase or decrease as a function of trial number, but rather fluctuated around the mean value for both the 1 mV_4 and 1 mV_10 control blocks.

### 3.3. SICI Blocks

**Figure 4:**
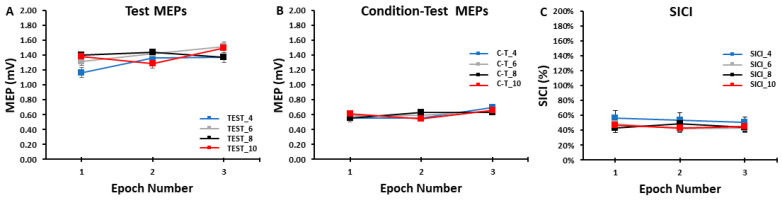
The test MEP, condition–test MEP, and SICI values obtained in the SICI trial blocks. There were no significant differences between the 4, 6, 8, and 10 s ITIs and across the three epochs for the test MEPs (**A**), the condition–test MEPs (**B**), or SICI (**C**).

**Figure 5:**
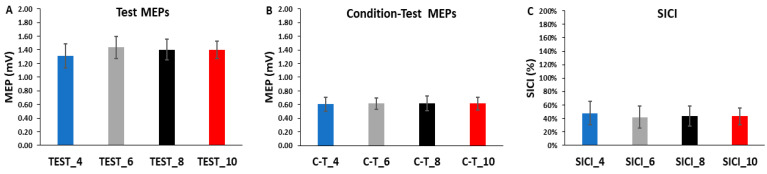
The test MEPs, condition–test MEPs, and SICI magnitudes for the 4, 6, 8, and 10 s ITIs (**A**–**C**) are shown as the overall averages for each SICI trial block. There were no significant differences between ITIs for the test MEPs, the condition–test MEPs, or SICI.

The differences in test MEP amplitudes were compared across ITIs and epochs in the SICI blocks with a 4 *ITI* (TEST_4, TEST_6, TEST_8, TEST_10) × 3 *Epoch* (1, 2, 3) within-subjects ANOVA. The main effect for *ITI* (*p* = 0.861, η_p_^2^ = 0.011), the main effect for *Epoch* (*p* = 0.293, η_p_^2^ = 0.053), and the *ITI* × *Epoch* interaction (*p* = 0.477, η_p_^2^ = 0.039) were all non-statistically significant (Figure 4A and Figure 5A).

The differences in condition–test MEP amplitudes were compared across ITIs and epochs in the SICI blocks with a 4 *ITI* (C-T_4, C-T_6, C-T_8, C-T_10) × 3 *Epoch* (1, 2, 3) within-subjects ANOVA. The main effect for *ITI* (*p* = 0.999, η = 0.000), the main effect for *Epoch* (*p* = 0.194, η = 0.075), and the *ITI* × *Epoch* interaction (*p* = 0.683, η = 0.024) were all non-statistically significant (Figure 4B and Figure 5B).

The differences in SICI values were compared across ITI and epochs in the SICI blocks with a 4 *ITI* (SICI_4, SICI_6, SICI_8, SICI_10) × 3 *Epoch* (1, 2, 3) within-subjects ANOVA. The main effect for *ITI* (*p* = 0.127, η_p_^2^ = 0.082), the main effect for *Epoch* (*p* = 0.568, η_p_^2^ = 0.022), and the *ITI* × *Epoch* interaction (*p* = 0.836, η_p_^2^ = 0.013) were all non-statistically significant (Figure 4C and Figure 5C).

**Figure 6:**
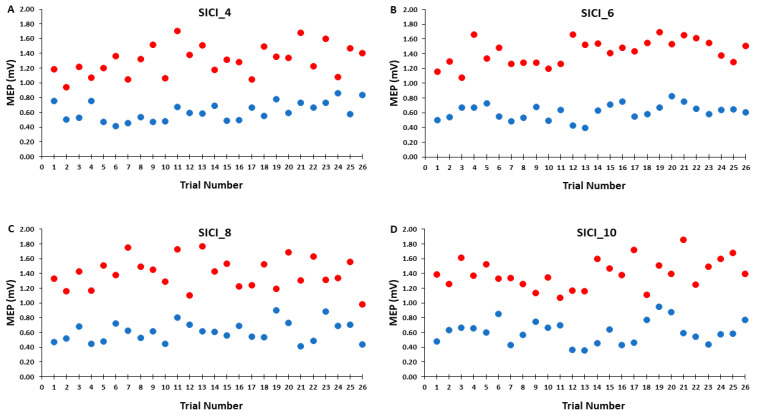
The test MEP (red) and condition–test MEP (blue) magnitudes across trials for the 4, 6, 8, and 10 s ITIs (**A**–**D**) in the SICI trial blocks are shown for illustrative purposes. Each data point corresponds to the average MEP amplitudes of all participants for each trial.

**Figure 7:**
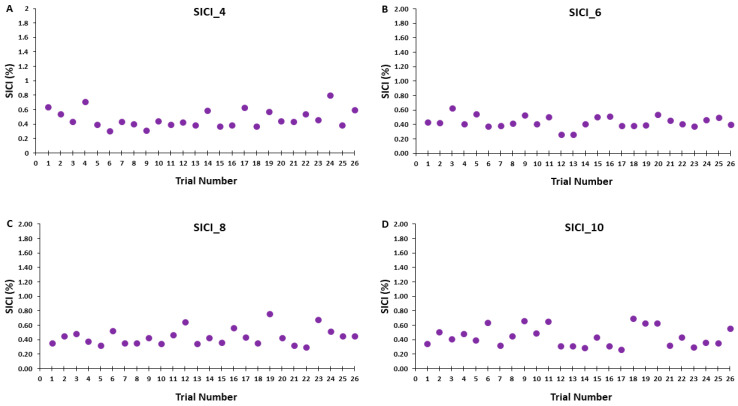
SICI magnitude across trials for the 4, 6, 8, and 10 s ITIs (**A**–**D**) are shown for illustrative purposes. Each data point corresponds to the average SICI of all participants for each trial in a SICI trial block.

Accordingly, Figure 6A–D illustrates that the test MEP amplitudes and condition-test MEP amplitudes did not show a trend for an increase or decrease as a function of trial number, but rather fluctuated around the mean value for the SICI_4, SICI_6, SICI_8, and SICI_10 blocks. Similarly, Figure 7A–D illustrates that the SICI values did not show a trend for an increase or decrease as a function of trial number, but rather fluctuated around the mean value for the SICI_4, SICI_6, SICI_8, and SICI_10 blocks.

## 4. Discussion

The primary purpose was to examine the effects of ITI on the quantification of SICI at rest. The secondary purpose was to determine the effects of ITI on the quantification of single-pulse MEP amplitudes at rest. The main findings were as follows: (1) The single-pulse MEP amplitudes in the 1 mV_4 and 1 mV_10 conditions did not display serial reductions with time in either of these control blocks. Accordingly, the overall average MEP amplitudes did not differ for the 1 mV_4 and 1 mV_10 blocks. (2) The magnitude of SICI did not exhibit a serial increase (greater inhibition) with time for any of the four ITIs (4, 6, 8, and 10 s) that were investigated in the SICI blocks. Similarly, the overall average SICI values were also not different between the four SICI blocks. Collectively, these results indicate that 4 and 10 s ITIs give similar overall single-pulse MEP values and 4, 6, 8, and 10 s ITIs give similar SICI values. Therefore, ITI had no meaningful influence on single-pulse MEP amplitude in the control blocks or on the test MEP, condition–test MEP, and therefore SICI values obtained under the current experimental conditions.

### 4.1. The Influence of ITI on MEP Amplitudes in the Control Blocks

The MEP elicited by TMS applied over M1 provides a simple measure of corticospinal excitability at the time of arrival of the TMS pulse. MEPs are widely used to understand the physiological mechanisms responsible for producing and controlling movements. Although MEPs can be evoked and recorded relatively easily, numerous methodological issues and experimental controls are needed to obtain valid MEP measurements in specific experimental circumstances. Accordingly, numerous review articles have focused on the optimal TMS parameters to use when obtaining single and paired-pulse MEPs [29,61,62]. However, ITI has received much less little attention in comparison to other TMS parameters involved in single-pulse TMS, but especially in regard to paired-pulse TMS. It could be that ITI has been inadvertently neglected or perhaps this can be attributed to the prevailing notion that there are no post-stimulation effects at the most commonly used ITIs. Nonetheless, well over a decade ago, a significant majority of an international assembly of TMS experts emphasized the importance of controlling and reporting ITI in TMS experiments, despite the paucity of direct systematic investigations available at that point in time [21]. Contrary to these suggestions, the vast majority of TMS studies rarely mention or explicitly report ITI information in their methods sections. This observation is substantiated by a TMS review where, among 16 the reviewed studies, only 1 (~6%) disclosed the ITI employed [30]. This underscores that ITI lacks a unified approach, which is further evidenced by the varied ITI ranges and methods for adjusting ITI across trial blocks documented in existing literature.

The present study included two control blocks performed under resting conditions to examine the effects of short (4 s) and long (10 s) ITIs on single-pulse TMS MEP amplitudes (1 mV_4 and 1 mV_10 conditions, respectively). These blocks were performed to provide control measures for both the test MEPs completed in the SICI blocks and for the overall measurement of SICI. The control blocks served the dual purpose of being a control comparison to the single-pulse test MEPs evoked in the SICI blocks and enabling the findings to be compared to previous single-pulse ITI studies [35,36,37,39,41]. In general, the main findings of these studies were similar and reported MEP amplitude suppression of approximately the first 10 MEPs of a block of 20–30 trials when ITIs of less than or equal to 5 s were administered. This issue could therefore be a potential confound in any previous or future studies involving MEPs evoked with ITIs in this range.

Consequently, the a priori hypothesis of the current study was that the 1 mV_4 control block would exhibit a lower overall MEP amplitude than the 1 mV_10 block due to MEP suppression in approximately the first 8–10 trials. However, the results were not consistent with this hypothesis as there were no differences in overall block average between the two control blocks. Furthermore, MEP amplitudes in epoch 1 were also nearly identical compared with epochs 2 and 3 for both control blocks, which provides no evidence of a serial reduction in MEP amplitude with time in these conditions (Figure 2A,B). In addition, visual inspection of Figure 3A,B not only reflects these statistical outcomes, but also clearly shows no discernable pattern in regard to the first MEP trial being larger in amplitude than subsequent MEPs later in the block. Accordingly, the first 1–5 MEPs were squarely in the middle of the range of variation of MEP amplitude observed across the entirety of the control blocks.

Taken together, the current results are in contrast to the majority of the findings in prior ITI single-pulse TMS studies. At first glance, these inconsistencies appear rather difficult to explain, but a detailed examination of the details and nuances of or each study points to sample size and methodological disparities as the most likely explanations. In regard to sample size, the maximum number of participants was 17 and the average was 12.5 across these investigations [35,36,37,39,40,41]. This is important as sample size is the factor that provides the largest mathematical contribution to the estimator error for MEP amplitude quantification [42].

A good example of a methodological difference is a study by Moller and colleagues [36], which reported that recruitment curves determined with a 5 s ITI were significantly different compared to an ITI of 20 s. A key issue, however, was that a wide range of TMS intensities are used when obtaining recruitment curves compared to the constant simulation intensities employed in this study, previous ITI studies, and in the majority of TMS blocks in most experimental conditions. Additionally, that study only collected five MEPs per block, a number far under the 20–30 recommended to minimize MEP error estimation [42]. In another study that involved only nine participants, a 2 s ITI resulted in significant lower MEPs compared with ITIs of 5 and 10 s [39], which is only partially consistent with the current results. In a much more comprehensive study, MEPs were lower at short ITIs of 1, 2, 3, and 5 s when compared to a long 10 s ITI. This finding was mainly due to the first 10 MEPs collected in blocks consisting of 30 total trials. Importantly, this study was characterized by several structural and procedural differences in comparison to the current study. First, the sample size comprised eight total participants. Second, biphasic TMS pulses were utilized compared with the much more common monophasic pulses used in most single pulse TMS studies, a difference that is well-known to yield different MEP results [63]. Third, the participants were allowed to watch television during the experiments versus the common practice of monitoring visual EMG feedback of muscle activity or having no significant external stimuli. Because MEP amplitude can be different within the up and down phases of neuronal oscillations as quantified by EEG and by attention level [6], this experimental issue could have significantly influenced results. A study by Schmidt and colleagues [40] has also provided results in opposition to the current ones. These authors reported that an ITI of 3 s produced a temporary initial state where MEP amplitudes in the first 20 trials were significantly lower compared with subsequent trials. One major difference in this study versus other ITI studies was that ITIs of over 3 s were not investigated, which makes meaningful comparisons with our study difficult. On the other hand, another report with very similar methodology to the many aspects of the single-pulse portion of the present study indicated that MEPs were significantly reduced in a 4 s as opposed to a 10 s ITI condition [41]. A second study by the same authors using similar methods indicated that MEP amplitudes at an ITI of 5 s were lower compared with 10, 15, and 20 s ITIs. In these two cases, the possible explanations for divergent findings compared to the current are somewhat difficult to reconcile.

In contrast, the current results align with a seminal repetitive TMS study [33], which discovered that an ITI of one second did not affect successive MEPs, indicating that ITIs shorter than one second were needed to induce significant after-effects on MEP amplitude. This conclusion is bolstered by physiological findings showing that a solitary TMS pulse applied to M1 only elevates cortico-muscular coherence for a duration of 300–800 ms before returning to baseline levels [34]. Furthermore, our recent study that involved the influence of ITI on ICF was conducted in nearly identical experimental conditions as the current study and also found no evidence of time-dependent effects in the single-pulse TMS control blocks [38]. To conclude, the overall literature displays mixed findings on the effect of brief ITIs of 5 s or less on MEP amplitude measurement with the preponderance of the available studies being in opposition to the present results. However, differences in methodology and the much lower sample sizes in previous studies likely account for most of these discrepancies.

### 4.2. The Influence of ITI on the Measurement of SICI

SICI is the most common and extensively studied paired-pulse TMS protocol [7,10,11] Although a series of previous studies by different research groups have investigated the effect of short and long ITIs on single-pulse TMS amplitudes, this was the first study to examine the effects of ITI on the measurement of SICI. Based on these prior single-pulse studies [35,36,37,39,40,41], the a priori hypothesis of the current study was that the magnitude of SICI in the short (4 s ITI) SICI block would be increased (greater inhibition) compared with the 6, 8, and 10 s ITI SICI blocks. In addition, it was hypothesized that these differences would be manifested through an initial suppression (greater inhibition) over approximately the first 8–10 MEP trials and would not be due to a serial increase (more inhibition) in SICI over the entire trial block. Theoretically, some weighted contribution of both of these elements within SICI_4 block relative to the to three SICI blocks with longer ITIs could also occur. Any of these results would imply that prior SICI studies that utilized ITIs of 4–5 s or less may have provided imprecise or even partially inaccurate results.

A total of 52 MEP trials were attained in each of the four SICI blocks. Each block comprised 26 test MEP trials and 26 condition–test MEP trials presented in a semi-random fashion. For analysis, these sets of responses were divided into time three epochs and were collectively utilized to calculate SICI. Separate analyses were also performed on the test MEP trials and condition–test MEP trials alone to examine their individual role in determining the magnitude of SICI induced in each of the four SICI blocks. For the test MEP trials alone, the results mirrored those of the control blocks as there was no indication of a serial reduction in MEP amplitude over any of the epochs of test MEP trials in any of the SICI blocks (Figure 4A). Therefore, the overall average test MEP was also not different between the four SICI blocks (Figure 5A). All of these statistical findings are clearly corroborated by the graphical representation of the group average test MEP amplitudes plotted by trial number (Figure 6A–D). In conclusion, the test MEP amplitudes when analyzed alone displayed no differences across epochs or between SICI blocks.

Similarly, the condition–test trial MEP amplitudes when analyzed alone displayed the exact pattern of findings and therefore overall results as the test MEP trials. They failed to exhibit time-varying characteristics over any of the epochs that comprised the total of 26 condition–test MEP trials in any of the four SICI blocks (Figure 4B). Accordingly, the overall average condition–test MEP was also not different between the four SICI blocks (Figure 5B). Thus, condition–test MEP amplitudes randomly varied around the average value obtained over the entire duration of the SICI blocks. All of these statistical findings are clearly supported by the graphical representation of the group average condition–test MEP amplitudes plotted by trial number (Figure 6A–D). In conclusion, the condition–test MEP amplitudes when analyzed alone displayed no differences across epochs or between SICI blocks.

Since the test MEP and condition–test MEP are the two components that comprise the measurement and mathematical calculation of SICI, the same overall pattern of results had to manifest for SICI. Therefore, there was neither a serial increase (greater inhibition) in SICI over the course of any of the SICI blocks (Figure 4C) nor a difference in the overall SICI block average values (Figure 5C). These findings were reflected in the graphical representations of the group average SICI values plotted as a function of trial number (Figure 7A–D). Thus, there were no significant differences in the magnitude of SICI obtained across epochs or between SICI blocks. In summary, the present findings were relatively straightforward as ITI had no meaningful influence on single-pulse MEP amplitude in the control blocks or on the test MEP, condition–test MEP, and therefore SICI values obtained under the current experimental conditions.

### 4.3. Methodological Considerations

There are numerous methodological issues that can potentially impact the quantification of single- and paired-pulse MEPs. Thus, this study implemented nearly all the elements of methodological quality that have been proposed for single- and paired-pulse TMS studies [6,21,30]. In addition, the study employed similar procedures to most previous single-pulse TMS studies that explicitly studied the influence of ITI on MEP amplitude [35,36,37,39,40,41]. The study was also limited to healthy young adults and had nearly equivalent numbers of women and men. Therefore, any differences that could have occurred due to any disorder, a wide age range, or an unequal distribution of participants by gender was greatly minimized. The possible influence of handedness or degree of laterality was also likely very low as all participants were strongly right-handed. Lastly, MVCs were also performed before and after the main aspect of the experiment to verify that the target FDI muscle’s voluntary activation levels did not significantly change over the entire course of the experiment. Although the experiments were performed completely at rest it may have been possible that concentration, arousal, or alertness [6] could have decreased during that rather long experiment and impacted MEP amplitude.

The data analysis aspect most pertinent to the interpretation of results of the present study could be argued to be the somewhat common procedure to exclude the first few (e.g., 1–5) MEPs from the analysis of each trial block. However, close scrutiny of Figure 3, Figure 6 and Figure 7 all unambiguously show that this procedure would not have influenced any of the current findings. It appears that the first mention of this method of MEP data reduction was in a study dating back to the earliest days of TMS [29]. The authors stated that they usually excluded the first MEP because it displayed a greater amplitude compared with all the successive MEPs of the trial block. In that study, the ITI employed in that study was randomly given within a range of 3.5 and 7 s. However, this appeared to be based solely on subjective examination and no objective calculations were provided. Another possible origin to the idea of deleting the first several MEPs was a fatigue study [61] that reported a serial decline in the initial four MEPs of trial block. Nevertheless, the condition of the motor system and corticospinal excitability subsequent to the completion of a fatiguing contraction is not comparable to rest and the evolution of the after-effects of fatigue obviously contributed to those results. Accordingly, a comprehensive study that focused only on the effects of initial MEP removal concluded as long as an adequate number of total MEPs were collected the deletion of the first 3–5 MEPs did not significantly influence results [64].

### 4.4. Practical Applications for SICI Studies

The current results have a number of practical implications for single-pulse TMS studies and the quantification of SICI. First, the tactic of removing the first MEP or initial several MEPs of a trial block from analysis seems to be an unwarranted loss of data and not necessary. Second, the widespread approach of adjusting the SICI test MEP amplitude to 1 mV or slightly higher was further supported in the current study for ITIs ranging between 4 and 10 s and appears to be the best practice. By extension, the less frequent method of setting the test MEP stimulation intensity to 110–120% of the RMT is also viable as this almost always elicits MEPs of 1 mV or slightly [42]. Third, it probably does not matter if short and long ITIs are employed in a randomized fashion within the same block of trials as long as they are in the range of 4 to 10 s. Therefore, some researchers may want to do this in experimental circumstances where they do not want the participants to anticipate the TMS pulses that would occur at constant fixed intervals. Fourth, the administration of the 10 s ITI condition clearly indicated to the experimenters and participants that ITIs of that length or longer are uncomfortable, time-inefficient, and not necessary [37] to obtain the same results as shorter ITIs. In summary, it is recommended based on the current findings that ITIs of between 4–6 s represent the optimal balance between reasonable MEP amplitude estimation, participant comfort, and the use of the time of investigators. Obviously, this approach may be especially relevant to older adult or patient populations who may find it more difficult to undergo prolonged experiments relative to younger adults. These recommendations seem to be congruent with the calculations of a recent review article [42], which gave mathematically derived guidelines for determining the trade-offs involved when considering the interrelated experimental variables of sample size, MEP variability and error estimation, experiment time, MEP trials per block, and the total number of blocks.

### 4.5. Limitations

The study had a few potential limitations that warrant discussion. First, the study only used a single constant ITI in all trial blocks. Many studies have chosen to randomly present MEP trials of different ITIs with a range of several seconds within blocks. However, it is pretty unlikely that varying ITI over a span of several seconds would have resulted in different overall results based on the current findings and prior studies [35,36,37,39,40,41]. Nonetheless, a comparison between constant and varied ITIs could be warranted in subsequent studies. Second, only one set of stimulation parameters were used to evoke SICI. This set was carefully chosen to reflect not only the most frequent in the literature, but also the best to produce the greatest SICI [12]. Despite these considerations, it cannot be completely discounted that a different set of stimulation intensities and ISI could deliver dissimilar findings as modification of some of these parameters provide measurements of SICI that are due to different populations of intracortical neurons [13]. Third, the single-pulse control blocks and the SICI blocks did not investigate ITIs below four seconds. However, as mentioned previously this was often not possible due to the TMS device’s limitations for SICI. Although this could have been carried out for the single-pulse blocks [35,36,37,39,40,41], this approach would have not fit well into our research design for comparisons to the SICI measurements and would have been a repetition of several of the aforementioned previous studies. Fourth, none of the TMS measures in the current study were conducted under experimental conditions involving FDI muscle contraction. On the other hand, former studies have clearly shown that ITI has influence on MEP quantification during active muscle contraction [36,39] due to the fact that the values of background cortical excitability and therefore EMG levels are relatively stable compared with the fluctuating levels of cortical excitability over small time scales at rest. Fifth, the other possible pathways measured with paired-pulse TMS such as ICF, LICI, and SICF were assessed in the current study. However, based on our previous similar ITI study involving ICF [38], which yielded a similar lack of influence of ITI on results, it is highly improbable that ITI would differentially influence these other paired pulse measures. Sixth, the number of participants in the study could be viewed as low as in many neurophysiology studies in general [65,66]. As mentioned above, however, the current sample size of was substantially greater that all former single-pulse ITI studies by a wide margin (grand average of 12.5 and maximum of 17 participants) in these investigations [35,36,37,39,40,41]. Finally, the effect sizes were exceedingly low in the current study and provided objective evidence that further increasing the sample size would be unlikely to change the results and to have been a worth the additional time and resources to accomplish.

## 5. Conclusions

The major findings indicated that measurements of SICI neither differed between ITIs ranging between 4 and 10 s nor demonstrated significant time-dependent amplitude changes within blocks of trials. MEPs elicited with single-pulse TMS exhibited analogous overall results between the ITIs and during trial blocks. Based on these two sets of findings, it appears that ITIs of 4–6 s provides comparable results for SICI relative to longer ITIs, while having the advantages of being more comfortable to participants and reducing the experimental time needed to evaluate perform single- and paired-pulse TMS experiments.

## Data Availability

The data presented in the study are available on request from the corresponding author.
